# Micropattern-based nerve guidance conduit with hundreds of microchannels and stem cell recruitment for nerve regeneration

**DOI:** 10.1038/s41536-022-00257-0

**Published:** 2022-10-20

**Authors:** DoYeun Park, Donghak Kim, Su Jeong Park, Jeong Ho Choi, Yoojin Seo, Dong-Hwee Kim, Sang-Hoon Lee, Jung Keun Hyun, Jin Yoo, Youngmee Jung, Soo Hyun Kim

**Affiliations:** 1 KU-KIST Graduate School of Converging Science and Technology, Korea University, 145 Anam-ro, Seongbuk-gu, Seoul, 02841 Republic of Korea; 2 Center for Biomaterials Research Center, Korea Institute of Science and Technology, 5 Hwarang-ro 14-gil, Seongbuk-gu, Seoul, 02792 Republic of Korea; 3 Institute of Tissue Regeneration Engineering, Dankook University, Cheonan, 31114 Republic of Korea; 4 Center for BioMicrosystems, Brain Science Institute, Korea Institute of Science and Technology, 5 Hwarang-ro 14-gil, Seongbuk-gu, Seoul, 02792 Republic of Korea; 5 School of Biomedical Engineering, College of Health Science, Korea University, 145 Anam-ro, Seongbuk-gu, Seoul, 02841 Republic of Korea; 6 School of Electrical and Electronic Engineering, Yonsei University, Seoul, 03722 Republic of Korea; 7 Korea Institute of Science and Technology (KIST) Europe, Campus E 7.1, 66123 Saarbrücken, Germany

**Keywords:** Regenerative medicine, Biomaterials, Tissue engineering

## Abstract

Guiding the regrowth of thousands of nerve fibers within a regeneration-friendly environment enhances the regeneration capacity in the case of peripheral nerve injury (PNI) and spinal cord injury (SCI). Although clinical treatments are available and several studies have been conducted, the development of nerve guidance conduits (NGCs) with desirable properties, including controllable size, hundreds of nerve bundle-sized microchannels, and host stem-cell recruitment, remains challenging. In this study, the micropattern-based fabrication method was combined with stem-cell recruitment factor (substance P, SP) immobilization onto the main material to produce a size-tunable NGC with hundreds of microchannels with stem-cell recruitment capability. The SP-immobilized multiple microchannels aligned the regrowth of nerve fibers and recruited the host stem cells, which enhanced the functional regeneration capacity. This method has wide applicability in the modification and augmentation of NGCs, such as bifurcated morphology or directional topographies on microchannels. Additional improvements in fabrication will advance the regeneration technology and improve the treatment of PNI/SCI.

## Introduction

The goal of tissue engineering and regenerative medicine is to regulate cell alignment in tissues within a compatible environment. Especially for nerve injuries, guided reconnection of two nerve stumps in a regeneration-friendly condition is critical for enhanced regeneration. Nerve guidance conduits (NGCs) are used for peripheral nerve injuries (PNIs) to avoid life-long disability in 200,000 and 300,000 surgeries in the US and Europe, respectively^1,2^. Autografts/allografts are regarded as the gold standard^3^. The application of autografts/allografts is limited owing to their scarcity, and dimensional differences (size and geometry) compared to the injured site can lead to large injury gaps. In addition, inflammation caused by donor site resection can result in the formation of a neuroma^1,4^. Single-channel NGCs (sNGCs) isolate nerve fibers to improve their regeneration capabilities^5^. In some cases, stem cells are seeded into NGCs to provide a regeneration-friendly environment^6,7^. However, due to their limitations, outcomes were loss of function in subjects. Implanted stem cells can form a regenerative milieu but are a serious threat if they are not well regulated, with negative effects such as unexpected differentiation and malignant transformation after transplantation, in addition to ethical issues and safety concerns, such as quality assurance^8^. The use of sNGCs leads to poor functional regeneration^9^. As shown in Supplementary Fig. 1a, no spatial guidance for the regeneration of nerve fibers exists within the sNGC. The sNGC provides hundreds of regenerative nerve fibers with one path having much larger dimensions than the individual fibers. The fibers extend lateral buds in any direction to search for a target and pruning the buds when one of the buds reinnervates any target^10–12^. In other words, without spatial guidance, regeneration can very likely end with the fibers being reinnervated to the incorrect targets (i.e., poor functional regeneration). Therefore, size-tunable NGCs with multiple guidance channels (Supplementary Fig. 1b) and endogenous stem cells are required to achieve a high level of nerve regeneration.

Numerous advances in fabrication methods have been pursued to produce an optimal NGC that reduces the mismatch and inappropriate reinnervation. Methods such as electrospinning^13^, 3D printing^14,15^, and thermal drawing^16^ can control the overall NGC size. Fabrication methods have been modified and advanced to deform the inner surface of sNGC to better align nerve fibers^17,18^ or form a multichannel NGC (mNGC) with few channels^19,20^. Among these, micro-electromechanical systems (MEMS) are emerging as a suitable method for optimal NGC manufacturing. MEMS-based structures are tunable in the dimensions of millimeters to nanometers and can be patterned only polymer sheets as topographic cues for cell alignment^18,21–23^. Researchers have employed micropatterned poly(L-lactide-co-ε-caprolactone) (PLCL) sheets to promote nerve regeneration in the direction of the micropattern^18^ and have produced NGC using SU-8 and polydimethylsiloxane (PDMS) as the main constituents^20^. However, these NGCs have clear limitations; they are essentially sNGC, and the NGC constituents (SU-8 and PDMS) are unsuitable for implantation owing to the fact that they are not biodegradable and bio-inert^24^. Therefore, the MEMS-based fabrication of a biocompatible multichannel NGC has not yet been achieved.

Desirable materials for NGCs include collagen, polyglycolic acid (PGA), and PLCL, which are suitable owing to their biocompatibility and ease of use. In particular, PLCL is the main constituent of NEUROLAC^®^ (Polyganics, Netherlands), a clinically available NGC that has shown comparable regeneration to an autograft in some cases of rat motor function^25^. However, these materials alone cannot alter the regenerative environment of NGCs. Accordingly, various polymeric strategies—such as peptide immobilization have been pursued. Substance P (SP) is a neuropeptide known to be involved in cell proliferation, wound healing, neuronal differentiation, and the recruitment of endogenous stem cells^26–29^. Therefore, NGC with SP-immobilized PLCL may enhance the regenerative capability of nerve injury.

In this study, we used MEMS-based micropatterns and SP-immobilized PLCL for the fabrication of size-tunable NGCs with hundreds of microchannels and stem-cell recruitment capability for enhanced nerve regeneration. Multichannel NGCs (mNGCs) with various sizes of microchannels were fabricated to demonstrate the size tunability, and PC12 cells were employed to highlight the spatial guidance of the micropattern-based microchannels of mNGC. The mNGCs and mNGC with SP (mspNGC), affixed within an electrospun PLCL conduit, were implanted into an injured sciatic nerve of a rat (PNI) to guide the regrowth of nerve fibers. The progress of regeneration along the channels and stem-cell recruitment within mNGC and mspNGC were evaluated using immunofluorescence staining. The anatomical and functional recovery of the implanted rat was assessed by calculating the gastrocnemius muscle density and sciatic function index (SFI). Finally, to assess application in SCI model and structural modification, the mNGCs and mspNGCs were implanted into a completely transected T9-T11 spinal cord (SCI), and mNGCs with complex architectures such as bifurcated or topologically cued microchannels were fabricated for enhanced regeneration of tissues that underwent various forms of nerve injury.

## Results

### Fabrication and characterization of mNGC with stem cell recruitment capability

**Fig. 1:**
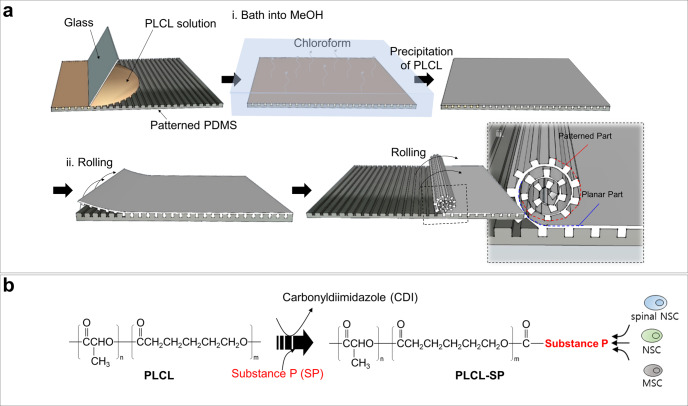
Production of a multichannel NGC displaying stem-cell recruitment ability. **a** Schematic diagram of the micropattern-based multichannel NGC fabrication process. PLCL solution was cast on patterned PDMS with dimensions (ridge width × ridge height × groove width) of 10 μm × 10 μm × 20 μm, 50 μm × 30 μm × 30 μm, and 100 μm × 50 μm × 30 μm, and spread using glass (i). The coated PLCL on the PDMS was bathed in methanol for precipitation from chloroform. PLCL holding on to the residual solvent acted as an adhesive, allowing the PLCL to roll. When the PLCL was rolled, the patterned part was attached to the planar part (ii). **b** Schematic diagram of SP immobilization on the PLCL molecules for stem-cell recruitment.

**Fig. 2:**
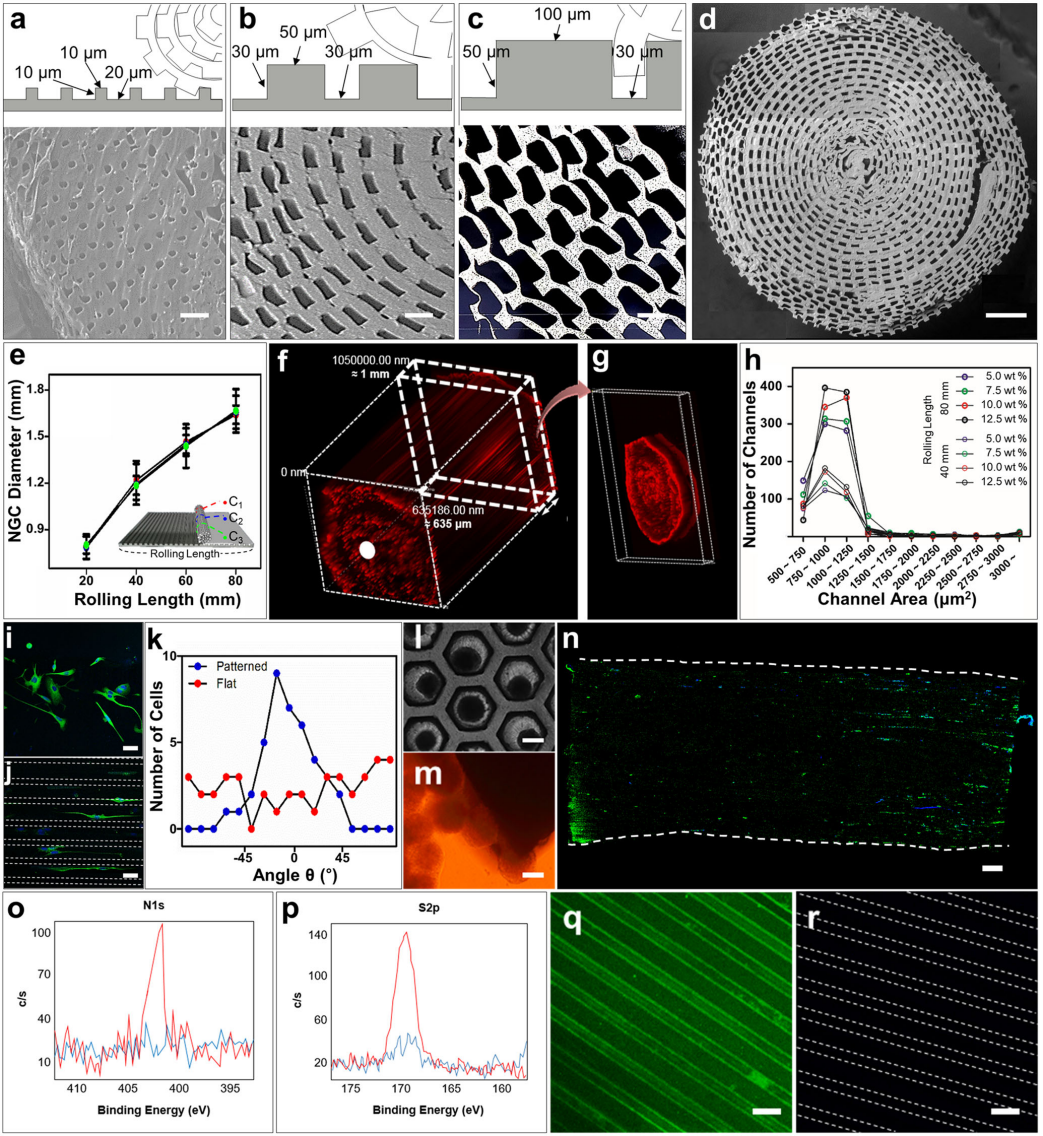
Size-tunability of the produced NGC and prototype selection. **a**–**c** Schematic diagrams (top) and SEM images (bottom) of various NGC micropatterns with dimensions (ridge width × ridge height × groove width (μm)) of **a** 10 × 10 × 20, **b** 50 × 30 × 30, and **c** 100 × 50 × 30. **d** SEM image of the s-section of NGC with a diameter 1.6 mm. **e** NGC diameter along with the rolling length (*n* = 3). Values are presented as mean ± SD. **f**, **g** Fluorescence images of the microchannels of the NGCs filled with red fluorescent polystyrene beads. **h** Channel-area distribution in mNGCs produced with different rolling distances (40 and 80 mm) and different concentrations PLCL solution (5.0, 7.5, 10.0, and 12.5%). **i**, **j** Fluorescence images of the PC12 cells cultured on **i** flat and **j** unrolled PLCL-patterned sheets. **k** The number of cells with a certain alignment angle between the micropattern and neurite. **l**, **m** Optical images of PC12 cell spheroids **l** formed on the concave mold and **m** cultured close to the NGC planes. **n** Fluorescence image of neurite growth from spheroids into mNGC. **o**, **p** XPS intensity measured for detection of SP on PLCL-SP. **q**, **r** Immunostaining SP on the unrolled PLCL-patterned sheet: **q** presence of SP and **r** absence of SP. Scale bars are as follows. **a**–**c** 50 μm, **d** 250 μm, **i**, **j** 50 μm, **l**–**n** 250 μm, and **q**, **r** 50 μm. Green fluorescence represents β-III tubulin antibodies in (**i**), (**j**), and (**n**), and SP antibodies in (**q**) and (**r**).

A schematic diagram of the micropattern-based fabrication of the mNGC is shown in Fig. 1a. Micropatterns with repeated ridges and grooves were engraved onto the PDMS mold via photo- and soft lithography. The details of the fabrication conditions are as follows. Before spreading the PLCL solution, the PDMS mold was pre-coated with 4% (w/v) Pluronic F127 (PF127) dissolved in DW to avoid microbubble formation between the mold and sheet (Supplementary Fig. 2a). The precipitation time was set such that the shape of the patterns on the sheet could be maintained without collapsing, and the solution residue remained and acted as a glue between the patterned part and the planar part of the sheet. Hundreds of microchannels were formed owing to stable adhesion between the patterned part and the planar part. Furthermore, during the experiment, the microchannel constructs of the multichannel NGCs were maintained without separation of the sheets. The ideal bathing time varied depending on the composition of the PLCL. For (5:5) PLCL, 10–20 min of bathing still resulted in microchannel collapse (Supplementary Fig. 2b), whereas less than 10 s of bathing for (7:3) PLCL resulted in complete precipitation, and this in turn could not form microchannels or maintain the NGC shape (Supplementary Fig. 2c). Therefore, we used a 50:50 mixture of (5:5) PLCL and (7:3) PLCL rather than only either, with a bathing time of 1 min. To provide the NGC with stem-cell recruitment capability, we immobilized SP onto PLCL molecules using CDI, as in Fig. 1b. The mspNGC was composed of PLCL (5:5), PLCL (7:3), and PLCL-SP (5:5). As illustrated in Fig. 2a–c, various sizes of micropatterns were produced (width of ridge × height of ridge × width of groove) to fabricate NGC, including 10 μm × 10 μm × 20 μm (Fig. 2a), 50 μm × 30 μm × 30 μm (Fig. 2b), and 100 μm × 50 μm × 30 μm (Fig. 2c). Among the various types of NGCs, prototypes were selected for further experiments. mNGCs with dimension of 100 μm × 50 μm × 30 μm were excluded because adhesion between layers did not occur when the side of NGCs was confirmed by SEM after rolling. Two types of NGCs with microchannel constructs with width × height × neighboring distances of 10 μm × 10 μm × 20 μm (Supplementary Fig. 2d) and 50 μm × 30 μm × 30 μm (Supplementary Fig. 2e) with 1.6 mm diameter were implanted into the rat PNI to assess the growth of the nerve fibers in the microchannels. Because nerve fibers were not observed to be inward in the former case, the latter case was selected as the prototype (Fig. 2d). The NGC length was set by the width of the PDMS mold (12 mm).

Microchannels of the prototype NGC were well-formed without clogging. The diameter of the NGC increased as the PLCL sheet was rolled, but no significant difference in diameter was observed throughout the microchannel length (Fig. 2e). When one plane of randomly sectioned 1-mm-NGCs was exposed to a PBS solution with red fluorescent polystyrene beads, microchannels were filled up from the exposed plane (white dot in Fig. 2f) to the other plane (Fig. 2g) due to the capillary force. The maintenance of the channel shape depended on the final concentration of the mixed PLCL solution. NGCs were fabricated with PLCL concentrations of 5.0% (w/v), 7.5% (w/v), 10% (w/v), and 12.5% (w/v) to have diameters 1.2 mm and 1.6 mm, and the center region (noted as C2 in subset image of Fig. 2e) was cross-sectioned to count the number of microchannels within a certain section (Fig. 2h). Because using a higher concentration of the mixed PLCL maintained the microchannels in a good shape in the cross-sectional plane, 12.5% (w/v) mixed PLCL was selected for producing mNGCs. A concentration higher than 12.5% was too viscous to spread evenly on the pattern.

The microchannels we produced were employed to physically guide the alignment of PC12 cells in both 2D and 3D conditions. For the 2D condition, PC12 cells were cultured on an unrolled flat (Fig. 2i) or patterned PLCL sheet (Fig. 2j) and subjected to differentiation media for 12 days. These were immunostained (anti-β-III tubulin, green; 4,6-diamidino-2-phenylindole, dilactate (DAPI), blue) and visualized using fluorescence microscopy. The alignment angle (*θ*) between the pattern length direction and the longer axis of the cultured PC12 cell neurites was distributed narrowly around 0° when using the patterned PLCL but was widely distributed when using the flat PLCL (Fig. 2k). For the 3D condition, PC12 spheroids were formed within the PDMS microwells (Fig. 2l) and cultured close to the cross-sectional plane of the NGC (Fig. 2m). After 12 days in the differentiation media, the neurites grew parallel to the long axes of the microchannels (Fig. 2n). This result indicates that the proposed NGCs between two nerve stumps could improve the chance of forming reconnections to the original target via guidance for multiple neurons.

Measured by X-ray photoelectron spectroscopy (XPS), the intensity values of the N1s peak (Fig. 2o) and S2p peak (Fig. 2p) in PLCL-SP were higher than those in PLCL. This indicates the presence of nitrogen and sulfur in the PLCL-SP. In addition, immunostaining confirmed the presence of SP in the PLCL-SP patterned sheet (Fig. 2q) and its absence in the PLCL-patterned sheet (Fig. 2r).

### Nerve regeneration guided by NGCs

**Fig. 3:**
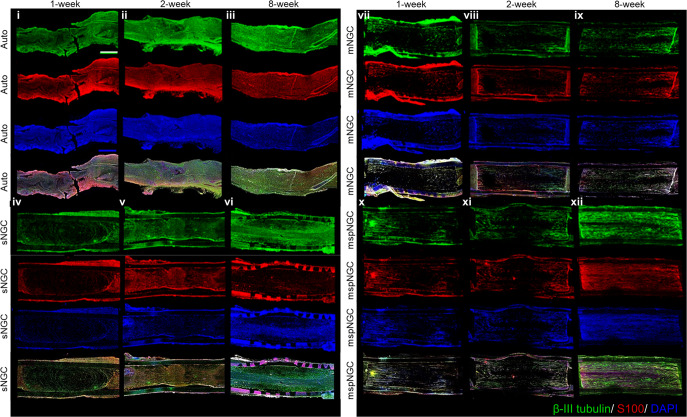
Nerve regeneration mediated by the produced NGC. Fluorescence micrographs of longitudinal sections of implant groups, showing neuronal regrowth along channels in **i**–**iii** autograft, **iv**–**vi** sNGC, **vii**–**ix** mNGC, and **x**–**xii** mspNGC. Green, red, and blue represent the expressions of β-III tubulin, S100, and DAPI, respectively. Scale bar indicates 1 mm.

Based on the aforementioned in vitro results, the proposed NGCs were implanted into the PNI, as illustrated in Supplementary Fig. 3, to assess the guidance of multiple microchannels for regenerating nerve fibers and the increased regeneration capability via stem-cell recruitment. Confocal images of the longitudinal-section planes of each group showed nerve regeneration within each NGC (Fig. 3). Compared with the autograft (implanting native tissue, Fig. 3i–iii) and electrospun sNGC (Fig. 3iv–vi), the guided reconnections of mNGC (Fig. 3vii–ix) and mspNGC (Fig. 3x–xii) were evaluated at weeks 1, 2, and 8 postoperatively in 12-weeks-old Sprague-Dawley rats. Confocal images at 12 weeks post-operation could not be obtained because of the collapse of microchannel constructs by degradation in mNGC and mspNGC during cryosection. Immunostained neurons filled the sNGC, mNGC, and mspNGC channels completely (green in Fig. 3). However, 1-week post-operation, nerve fibers from the central part of the tissue attached to the sNGC surface and regrew without any aligned pathways (Fig. 3iv). This observation indicated that, despite the fine anatomy observed for the rats at 8 weeks post-operation (Fig. 3vi), sNGC would likely lead to inappropriate target reinnervation^30^, attributed to the inherent features of nerve regeneration, such as lateral bud formation and pruning^17–19^. However, with mNGC or mspNGC, each of the hundreds of microchannels provides attachment surfaces to a few regenerating nerves to guide the reinnervation to the original target (Fig. 3vii, x). In addition to neuronal cells, generation of myelin by Schwann cells, an essential component of functioning neural tissue, was induced in the multiple channels of mNGC and mspNGC (Fig. 3ix, xi). The use of these NGCs can greatly decrease the correlation between incorrect innervation and the length of the injury or nerve location. Nerve fibers were observed to pass through the center of the mNGC (Supplementary Fig. 4a.ii) and mspNGC (Supplementary Fig. 4b.ii), reconnecting the two nerve stumps.

**Fig. 4:**
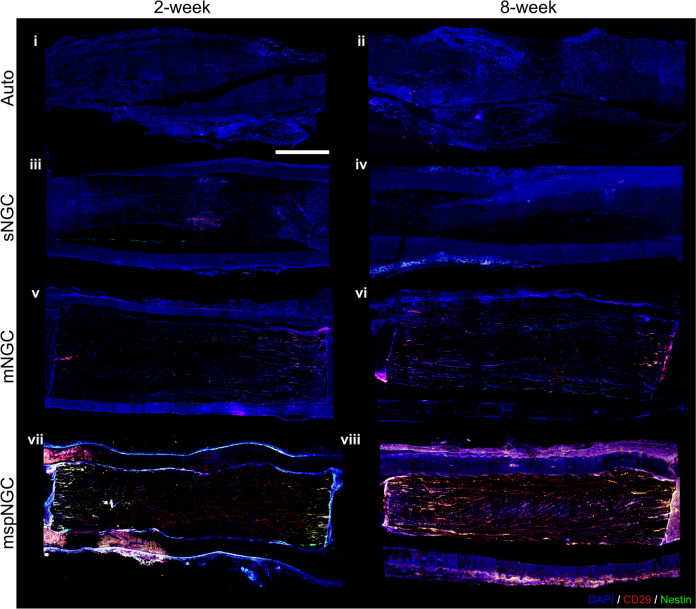
Recruitment of neural stem cells into NGC channels. Fluorescence micrographs of longitudinal sections of implant groups, showing neural stem/progenitor cell recruitment along the channels in **i**, **ii** the autograft, **iii**, **iv** sNGC, **v**, **vi** mNGC, and **vii**, **viii** mspNGC. Green, red, and blue represent the expressions of Nestin, CD29, and DAPI, respectively. Scale bar indicates 1 mm.

The mspNGC recruited endogenous stem cells with a high regenerative potential into multiple channels. As shown in Fig. 4, CD29 (stem-cell marker) and nestin (neural stem/progenitor cell marker) were expressed more in the mspNGC group than in any other group at 8 weeks post-operation.

**Fig. 5:**
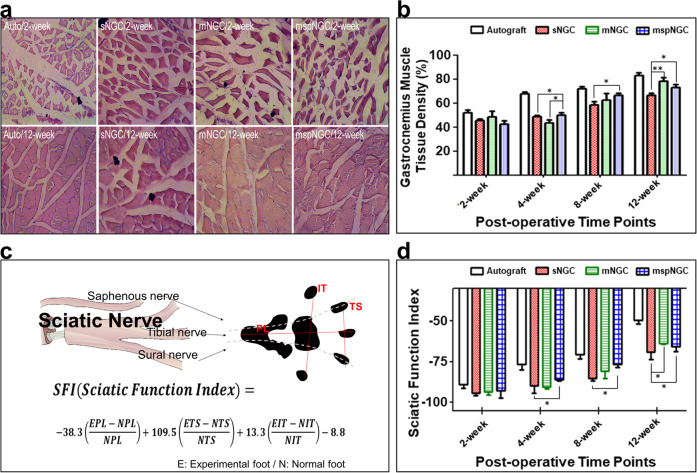
Evaluation of the functional recovery mediated by NGCs. **a** Optical images of H&E stained gastrocnemius muscle tissue of each group at various time points. **b** Gastrocnemius muscle tissue densities of each group at the indicated post-operative time points (*n* = 6 samples, *p < 0.05; **p < 0.01). **c** Schematic depiction of the SFI analysis. **d** SFI values of each implant group at post-operative time points (*n* = 4 samples, *p < 0.05). Values are *p*resented as mean ± SD.

To evaluate the functional recovery of the sciatic nerve, gastrocnemius muscle density and SFI were measured at 2, 4, 8, and 12 weeks postoperatively. Because muscle atrophy is restored when the denervated sciatic nerve is reinnervated (Fig. 5a), the gastrocnemius muscle density could be used as an indicator of reinnervation. As shown in Fig. 5b, reinnervation occurred in all groups according to muscle density measurements. No differences were observed 2 weeks postoperatively, but the recovery rate in the mspNGC group was remarkable at the beginning of recovery. At 4 weeks, the muscle density in the mspNGC group was the highest among the groups, and marked nerve innervation was observed in both mNGC and mspNGC groups at 8 and 12 weeks. SFI is an indicator of innervation quality. It is an index of motor functional recovery in rats and can indirectly measure the degree of innervation in each toe by analyzing its footprint using a well-known equation (Fig. 5c). Reversed autograft transplantation is the gold standard and supports the best nerve recovery. A gradual improvement in functional recovery was achieved following the introduction of NGCs. The two multichannel groups showed higher SFI than the single-channel groups, and the increase in SFI for the mspNGC group was particularly rapid at 4- and 8-weeks post-surgery (Fig. 5d). This is because of the higher probability of target reinnervation owing to multiple channels, as well as the enhanced nerve regeneration and neural stem/progenitor cell recruitment by SP. In addition, based on the observation of cross-sectioned SEM images for each group, the G-ratio indicating the maturation of nerve fibers in the mNGC and mspNGC groups was similar to that in the autograft group after 8 weeks (Supplementary Fig. 5b–f, g). Therefore, the mNGCs we produced are considered successful in guiding the regeneration of nerve fibers and recruiting stem cells, thereby greatly enhancing nerve regeneration after PNI. Lower recovery at 12 weeks post-operation was caused by the accelerated degradation of mspNGC^31–33^. PLCL is a degradable polymer that can be degraded in vivo via an enzymatic mechanism, physical stress, oxidation, and hydrolysis. As shown in Supplementary Fig. 6a, the molecular weight of NGC calculated by GPC decreased with time, and there was no significant difference in the molecular weight at 4- and 8 weeks; The molecular weights of sNGC, mNGC, and mspNGC were 72911 ± 7774, 74826 ± 9502, and 84448 ± 2793 g/mol at 4 weeks and 40413 ± 567, 38153 ± 4117, and 38850 ± 5750 g/mol at 8 weeks, respectively. However, mNGC and mspNGC were observed that have much lower molecular weights than sNGC at 12 weeks (Supplementary Fig. 6a, b), specifically 28401 ± 789, 19814 ± 19814 ± 328, and 19337 ± 137 g/mol for sNGC, mNGC, and mspNGC, respectively. In addition, to compare the mechanical properties of NGCs in Supplementary Fig. 6c–h, we performed tensile tests of the implanted NGCs post-surgery. The mechanical properties of NGCs were affected not only by the degradation rate but also by tissue recovery (Supplementary Fig. 6c–h). sNGC showed higher extension at 4 and 8 weeks, and higher strength at 4 weeks compared to mNGC and mspNGC. However, at 12 weeks, the mechanical properties of sNGC, mNGC, and mspNGC were low in all groups, and there was no significant difference.

### Applicability in the SCI model and structural modification

**Fig. 6:**
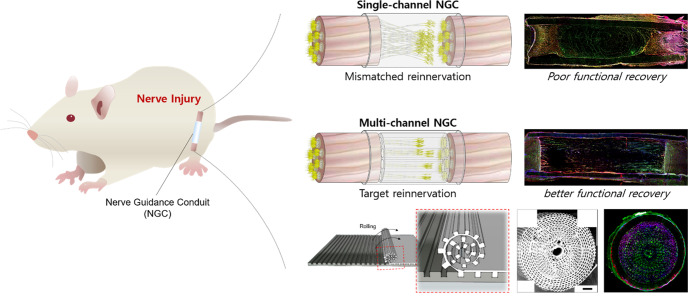
Summary figure. This paper deals with a micropattern-based method for fabricating size-tunable nerve guidance conduits (NGCs) with hundreds of microchannels capable of recruiting stem cells, with this recruitment ability derived from the properties of a substrate composed of substance P (SP)-immobilized PLCL (PLCL-SP). For functional recovery in peripheral nerve injury (PNI) and spinal cord injury (SCI), correct target reinnervation of nerve fibers is essential. However, hundreds of nerve fibers in single-channel NGCs cannot be reinnervated in place because of the extension of lateral buds on every side. In contrast, multichannel NGC maximizes functional recovery by minimizing mismatched reinnervation through a guide to the target fiber along the channel. In addition, it induces enhanced nerve regeneration by recruiting endogenous stem cells by substance P.

Our proposed NGCs can be used for other nerve injuries, including SCI regeneration (Fig. 6). The mNGCs and mspNGCs were implanted into SCI and compared with those without implants (Supplementary Fig. 7) using 12-weeks-old Sprague-Dawley rats. Two weeks post-operation, aligned nerve fibers were observed in both the mNGC group (Supplementary Fig. 8a) and the mspNGC group (Supplementary Fig. 8b). Compared with the mNGC group (Supplementary Fig. 8c), more stem cells were observed in the mspNGC group (Supplementary Fig. 8d). As mentioned above, appropriate target reinnervation by aligned nerve fibers can have a significant impact on functional improvement. Moreover, because the spinal cord contains multiple channels for neurons with different innervation targets and has repressed regeneration capacity^34^, we expect our NGCs to be an effective tool to reinnervation and improve regeneration in SCI, similar to PNI. In addition, mNGCs and mspNGCs could improve the regeneration of the damaged central nerve by suppressing the infiltration of reactive astrocytes that induce an inflammatory response (Supplementary Fig. 9). Activated astrocytes following injury have an inhibitory effect on neural development by inducing gliosis^35^. In a future study, we plan to conduct an in-depth evaluation of the potential of our NGCs to improve functional recovery and regeneration in SCI and to alleviate inflammatory responses by implantation.

Modification and augmentation of the proposed NGCs showed potential for regeneration in various nerve injuries. The overall morphology of NGCs can be manipulated. Several NGCs can be easily bonded to each other either entirely or partially (Supplementary Fig. 8e) or in a pyramid stack (Supplementary Fig. 8f) using chloroform residue on the NGC surface as a glue. This suggests its use in bifurcated nerve tissues (Supplementary Fig. 8g). Moreover, topographic cues were engraved on the surface of the microchannels (Supplementary Fig. 8h).

## Discussion

The nerve guidance conduit between two nerve stumps could improve the chance of forming reconnections to the original target via guidance for multiple neurons. In this paper, we developed a novel and simple method for fabricating NGCs with hundreds of size-tunable microchannels and with stem-cell recruitment capability using micropatterns and immobilized SP on PLCL molecules. To the best of our knowledge, this is the first study demonstrating the efficient fabrication of biomimetic multichannel NGCs containing hundreds of microchannels that actively enhance regeneration. We designed neural conduit fabrication to successfully control each dimension of NGC and microchannels. mNGCs were generated with various PLCL concentrations, precipitation times, and micropattern sizes, and we selected the condition in which the micropattern channels of the NGCs were well maintained and suitable for nerve growth within the NGCs—PLCL concentration (PLCL concentration of 12.5% (w/v)), precipitation time (10 s of bathing for 50:50 mixture of (5:5) PLCL and (7:3) PLCL), and micropattern size (microchannel constructs with width × height × neighboring distances of 50 μm × 30 μm × 30 μm). When using sNGCs, the likelihood of misaligned reinnervation was likely to increase with increasing length of the injury and the distance from the proximal to distal nerve end increased. In contrast, the appropriate target reinnervation by the guide of the mNGC and the mspNGC showed significant improvement of functional recovery, unlike sNGC induced incorrect reinnervation to the wrong target. Providing directional topographic cues to the microchannels enhances the control over cell alignment as the cell morphology and directional alignments change in response to topographic signals within microenvironments of microns to nanometer scales^36–38^. Therefore, the proposed NGCs can induce accurate reinnervation of branched nerves and enhance nerve regeneration by the alignment of cells.

Next, as bio-inert polymers such as PLCL and PGA alone cannot promote the regeneration environment of NGC, strategies such as immobilizing specific molecules onto the main materials^27,29,39^, or mixing collagen or decellularized extracellular matrix with the main material^40–42^ within the scaffold have been used to create a biocompatible and regeneration-friendly environment. In this study, to improve stem cell recruitment and neuronal differentiation, we mixed linear PLCL with SP-conjugated PLCL. Since a previous study reported that PLCL conduit with 9.6% PLCL-SP had the highest MSC recruitment capacity among PLCL conduit with 3.4%, 6.6%, and 9.6% PLCL-SP^39^, we selected the PLCL-SP concentration for mspNGC as 10%. We believed that mspNGC with 10% PLCL-SP could enhance MSC recruitment. At 8 weeks post operation, higher expression of stem cells was observed in mspNGC group than in any other group. This is because SP recruits endogenous neural stem/progenitor cells (NSPCs) from the periphery to the injury site for tissue repair^43^. The recruited NSPCs enhanced nerve regeneration through neurogenesis, assistance in myelin formation, secretion of several neurotrophic factors, and release of anti-inflammatory cytokines^44–46^. SP promotes proliferation of NSPCs and induces neuronal differentiation of NSPCs by activating the mitogen-activated protein kinase/extracellular signal-related kinase 1/2 (MAPK/ERK 1/2) signaling pathway by binding to the NK-1 receptor^26,29^. Therefore, mspNGC could enhance endogenous neurogenesis and guide nerve cells, as well as recruit numerous NSPCs to accelerate peripheral nerve regeneration in vivo.

Appropriate targeted reinnervation by aligned nerve fibers can have a significant impact on functional improvement. As expected, mNGC and mspNGC showed higher gastrocnemius muscle density and SFI than sNGC at 4 to 12 weeks. However, at week 12, nerve regeneration of mspNGC was slower than that of mNGC in functional recovery, and structural collapse of mNGC and mspNGC was observed. It was confirmed that the decomposition rates of mNGC and mspNGC were faster than that of sNGC at 12 weeks, which is expected to be due to the high exposed surface area to volume ratio of mNGC and mspNGC. Since mspNGC consisted of 10% star-shaped PLCL (Mn ≈ 42,000) and 90% linear PLCL (Mn ≥ 121,000), the mass loss of mspNGCs could be faster than that of sNGC and mNGC which were composed of only linear PLCL. The degradation rate of low molecular weight PLCL was faster than that of high molecular weight of PLCL^47^. Since the NGC collapse originated from degradation nullify the positive effects of stem cell recruitment at week 12, we speculated that it is difficult to expect a high recovery rate of mspNGC in terms of functional recovery after 12 weeks.

Although star-shaped PLCL with stem cell recruitment function has limited long-term efficacy owing to its faster degradation rate compared to linear polymers, further studies are being conducted to maximize the functions of the neural conduit by optimizing stem cell recruitment, the degradation rate, and mechanical properties. By improving the function of mspNGC, it is considered that mspNGC could be utilized not only in the PNI model but also in the SCI model. Since the spinal cord contains multiple channels for neurons with different innervation targets and their regenerative capacity is inhibited^34^, we expect our NGCs to be an effective tool to improve reinnervation and regeneration in SCI, similar to PNI. In addition, activated astrocytes following injury have an inhibitory effect on neural development by inducing gliosis^35^. In a future study, we plan to conduct an in-depth evaluation of the potential of our NGCs to improve functional recovery and regeneration in SCI and to alleviate inflammatory responses by implantation.

## Methods

### NGC group preparation

(5:5) PLCL and (7:3) PLCL were synthesized by ring opening polymerization using L-lactide (PURAC biochemical, Corbion), *ε*-caprolactone (A10299, Alfa Aesar), 1-dodecanol (126799, Sigma-Aldrich), and tin(II) 2-ethylhexanoate (S3252, Sigma-Aldrich) with molar ratio of 10,000:10,000:1:1 and 14,000:6000:1:1, respectively. The mixture was polymerized by heat at 150 °C for 24 h under vacuum. The polymer was dissolved in chloroform, precipitated by methanol, and dried in vacuum oven for 72 h. The synthesized polymer was analyzed using gel permeation chromatography (GPC, 1260 Infinity II). The autografts were reverse implants of native nerve tissue. The sNGCs were electrospun PLCL conduits fabricated as follows. A PLCL mixture (50:50 mixture of (5:5) PLCL, Mn ≈ 121,000, and (7:3) PLCL, Mn ≈ 127,000) at a final concentration of 12.5% (w/v) was dissolved in hexafluoro-2-propanol (TCI, Japan). The PLCL solution was injected from a syringe with a 21-gauge needle toward the collector (diameter: 1.6 mm) at a distance of 15 cm, passing through a 21 kV voltage field. The rotation rate of the collector was set to 100 rpm. mNGCs and mspNGCs were fabricated following the method described in Fig. 1. To evenly roll the precipitated sheet, a 31-gauge microneedle was used as the rolling core. After obtaining an mNGC with a diameter of 1.6 mm, 2 mm of lateral parts of the mNGCs or mspNGCs were cut off from both sides with a microtome blade (Leica Biosystems, Germany) immediately after 1 min of bathing in liquid nitrogen.

For further experiments, NGCs were prepared in various ways. They were cross-sectioned 2 mm from the two lateral ends (C1 and C3) and center (C2) to measure the diameter of each position. The cut NGC was then placed vertically on the scanning electron microscope (SEM) stage, followed by coating with gold nanoparticles. Images were captured using a portable SEM (G-2 pro Phenom, ThermoFisher, USA). Synthetic NGCs (sNGCs, mNGCs, and mspNGCs) were placed under a vacuum for 5 days to completely remove the chloroform residue, followed by sterilization using ethylene oxide gas both in vitro and in vivo. NGCs were then placed on the surface of a phosphate buffer solution (PBS) under a vacuum. The NGCs were then immersed in PBS after ~15 min and left there for 24 h to hydrate the channels. For in vitro use, subsequent coating with poly(D-lysine) (Sigma-Aldrich, USA) and laminin (Sigma-Aldrich, USA) was followed by exchanging PBS with the corresponding solutions. Note that the same procedures were performed on the unrolled PLCL-patterned sheet (2D condition). In contrast, for in vivo use, no additional coating after hydration was performed for implantation into the PNI (sNGC, mNGC, mspNGC) or SCI (mNGC, mspNGC).

### Immobilization of SP onto PLCL molecules

Star-shaped PLCL was produced by ring opening polymerization using L-lactide (PURAC biochemical, Corbion), *ε*-caprolactone (A10299, Alfa Aesar), tripentaerythritol (107646, Sigma-Aldrich), and tin(II) 2-ethylhexanoate (S3252, Sigma-Aldrich) with molar ratio of 4000:4000:1:1. Under vacuum and with vigorous stirring, the mixture was heated at 150 °C for 24 h. The produced star-shaped PLCL was dissolved in chloroform and precipitated by methanol, then dried in vacuum oven. GPC measurement was performed to determine the molecular weight of star-shaped PLCL. SP (97% purity, Peptron, Republic of Korea) was covalently immobilized onto a star-shaped PLCL (PLCL made of 50% L-lactide and 50% ε-caprolactone, Mn ≈ 42,000) using carbonyldiimidazole (CDI) chemistry^27,29^. First, a mass of 12.4 g of PLCL was dissolved in 70 mL dichloromethane (DCM, Sigma-Aldrich, USA), and a mass of 0.5 g of CDI (115533, Sigma-Aldrich, USA) was dissolved in DCM (10 mL) with stirring for 24 h. The two solutions were mixed and stirred for 24 h under N_2_ gas to obtain CDI-activated PLCL (PLCL-CDI). The PLCL-CDI was then precipitated by pouring the solution into excess methanol and drying it under vacuum conditions for 3 days. Next, 4 g of PLCL-CDI was dissolved in 40 mL DCM with stirring for 24 h under N_2_ gas, and a mass of 15.1 mg of SP was dissolved in 20 mL of dimethyl sulfoxide using a vortex for 12 h. The SP solution was added to the PLCL-CDI solution for performing the reaction to exchange CDI with SP, and the reaction mixture was stirred for 48 h under N_2_ gas. PLCL-SP was precipitated using excess methanol and dried under vacuum conditions for 5 days. The presence of SP was verified using XPS and immunostaining. XPS patterns of the spin-coated PLCL-SP were obtained using Al Kα radiation (1486.6 eV) (PHI 5000 VersaProbe) and were compared with those of the spin-coated PLCL in the presence of nitrogen and sulfur peaks. Immunostaining of the PLCL-SP patterned sheet was performed using SP antibodies (Santa Cruz, USA), as described below.

### Fabrication of micropattern-based mNGC with stem cell recruitment capability

After the PDMS mold was pre-coated with 4% (w/v) Pluronic F127 (PF127) dissolved in DW, linear PLCL (PLCL made of 50% L-lactide and 50% ε-caprolactone, Mn ≈ 121,000 and 70% L-lactide and 30% ε-caprolactone, Mn ≈ 127,000) dissolved in chloroform was spread evenly onto the mold, followed by precipitation to form a patterned PLCL sheet in a methanol bath for 1 min. The patterned PLCL sheet was rolled such that the sheet patterns met the plain part of the sheet, forming a microchannel. The number of microchannels in the NGC was equal to the number of the ridges on the PDMS mold. To provide the NGC with stem cell recruitment capability, we immobilized SP onto PLCL molecules using CDI, as mentioned above^27^. After synthesizing the star-shaped PLCL, CDI was attached to the end of the PLCL molecules. SP then replaced CDI to form PLCL-SP. Since the SP was attached to star-shaped PLCL, the ratio of PLCL-SP to (5:5) PLCL to (7:3) PLCL for the mspNGC was 1:4:5.

### Characterization of mNGC

The diameters of the mNGC were measured at 2 mm from the two lateral ends (C1 and C3) and the center (C2). For C1, an asterisk mark was engraved into the PDMS mold, outside of the pattern, to distinguish it from C3. Other images of the cross-section planes were obtained from C2 (Fig. 2). Microchannels of mNGC with ~0.6 mm diameter were filled with PBS with red fluorescent polystyrene beads (Invitrogen, USA) using a capillary force. One side of the mNGC was exposed to a droplet of dye solution for 30 min. Confocal Z-stack images were captured for perpendicularly standing mNGCs. ImageJ software was used to analyze the obtained images. The diameter was measured; the number of microchannels was counted with a certain range of area; and the angle between the micropattern and neurite of PC12 cells was measured.

### PC12 cell culture

PC12 cells (ATCC CRL-1721) were cultured on flat and unrolled PLCL-patterned sheets, and PC12 cell spheroids were cultured close to the two lateral planes of the mNGCs. PC12 cell spheroids were formed on concave microwell PDMS platforms (StemFit, Microfit, Republic of Korea). Immediately after culture, the culture medium was changed from proliferation media (RPMI media (HyClone, USA) supplemented with 5% fetal bovine serum (HyClone, USA) and 1% penicillin-streptomycin solution) to differentiation media (proliferation media supplemented with 10% horse serum (Sigma-Aldrich, USA) and 50 ng/mL of nerve growth factor (Sigma-Aldrich, USA)).

### NGC implantation

Since neuronal ingrowth and microchannel maintenance of mNGCs with micropattern dimensions of 50 μm × 30 μm × 30 μm were superior to those of the other groups (with a dimension of 10 μm × 10 μm × 20 μm and 100 μm × 50 μm × 30 μm), in vivo tests were evaluated using three experimental groups (sNGC, mNGC, and mspNGC, *n* = 4) with micropatterns dimension of 50 μm × 30 μm × 30 μm. The Sprague-Dawley rats (12-weeks-old; male; DBL) were anesthetized with 2–3% isoflurane (Forane; Choongwae Pharma, Seoul, Republic of Korea) using V-1 Tabletop with Active Scavenging (VetEquip). For PNI, 10 mm sciatic nerve defects were made 5 mm upstream from the point at which the sciatic nerve is divided into smaller nerves. For the autograft group (Supplementary Fig. 3a), the cut nerve tissue was implanted in reverse; the epineurium of the tissue was sutured with that of the implant. An electrospun conduit (sNGC) was implanted (Supplementary Fig. 3b), and the epineurium of the tissue was sutured to the wall of the sNGCs. The sNGCs acted as holders for the mNGC or mspNGC (right-side image in Supplementary Fig. 3). The mNGC or mspNGC held by the sNGC was implanted (Supplementary Fig. 3c); the epineurium of the tissue was sutured with the wall of the holder. In the PNI model, neuronal regeneration of NGCs was assessed at three-time points (1-, 2-, and 8-weeks post-surgery), and functional recovery of NGCs was assessed at four-time points (2-, 4-, 8-, and 12-weeks post-surgery). For SCI, laminectomy was performed at T9-T11 (5 mm) of the spinal cord, followed by an incision of the central line of the dura mater. A complete transection was induced by removing the entire tissue. While the control group had no implants, mNGCs, and mspNGCs with a length of 5 mm were implanted, resealing the dura mater to hold the implants (Supplementary Fig. 6). Neuronal regeneration and stem cell recruitment were assessed at 2 weeks to verify the applicability in the SCI model. Suturing of the implants in PNI and resealing in SCI were performed using a 10-0 nylon suture (ETHICON, USA), and suturing of the muscles and skins in both cases was performed using a 3-0 nylon suture (AILEE, Republic of Korea). The implantation protocols for PNI and SCI used in this study were approved by the Institutional Animal Care and Use Committee of the Korea Institute of Science and Technology (2018-090). After implantation, rats were housed at room temperature and constant humidity (45–50%) individually, with free access to food and water under 12 h of light/dark cycle.

### Cryosection and staining process

To obtain immunostaining images and hematoxylin and eosin (H&E) staining images, cryosections were created. After euthanization of SD rats by CO_2_ inhalation, samples were collected with some surrounding tissue and fixed in 10% formalin solution (ForBioKorea, Republic of Korea) for at least 1 day and immersed in 10% sucrose for 1 h, 20% sucrose for 1 h, and 30% sucrose for 24 h. Each sample was then immersed in the frozen section compound (FSC, Leica Biosystems, Germany) for a day. At that point, the FSC with the sample inside it was frozen using liquid nitrogen. For longitudinal confocal images, the sample was frozen parallel to the ground, and for cross-section images, the center of the frozen sample was cut and positioned perpendicular to the ground, following by freezing. The muscles for H&E staining were frozen after being placed perpendicular to the ground. Twenty-micrometer-thick cryosections were made for immunostaining (ThermoFisher, USA). After the FSC was removed from the sectioned samples and the samples were treated with Proteinase K (Dako, USA) and a 4% bovine serum albumin solution, the neurons and stem cells of the samples were stained with β-III tubulin (1:100, neuronal marker, T8578), nestin (1:100, neural stem/progenitor cell marker, MA1-110), and integrin beta 1 (CD29) (1:200, stem-cell marker, ab179471) primary antibodies diluted in 1% BSA at 4 °C, overnight. After washing thrice with 1 × PBS, the samples were incubated with the corresponding secondary antibody (1:1000, Alexa Fluor 488 IgG (A-11001) and Alexa Fluor 594 IgG (A-11012)) for 4 h at RT. The cells of samples were confirmed by the location of DAPI expression. In the case of PNI, Schwann cells were stained with S100 (1:100, Schwann cells marker, ab52642), followed by secondary antibody (1:1000, Alexa Fluor 594 IgG, A-11012). The expression of S100 antibodies was observed to surround the expression of β-III tubulin antibodies. In the case of CNI, astrocytes were stained with Glial fibrillary acidic protein (GFAP) (1:5000, astrocyte marker, ab7260), followed by secondary antibody (1:1000, Alexa Fluor 594 IgG, Invitrogen, A-11012) Cryosections for H&E staining were obtained, each with a thickness of 6 μm, and the sample was stained with hematoxylin and eosin for 13 min and 6 min, respectively.

### Functional recovery analysis

The gastrocnemius muscle tissue density, SFI, and G-ratio were calculated for the gastrocnemius muscle tissue density, and the ratio of tissue area to the whole area (tissue area + vacant area) was calculated. For the SFI value, each rat was passed through a transparent and straight acrylic passage at each time point (2-, 4-, 8-, and 12-weeks postoperatively, *n* = 4 for each time point). Footprints of the experimental and normal feet were captured from below. The SFI value was obtained by analyzing the PL, TS, and IT values, as shown in Fig. 5c. Finally, the G-ratio (axon diameter/(axon diameter + myelin thickness)) was calculated from the SEM images of the cross-sectioned NGC. This indicates the maturation of the regenerated nerve fibers. ImageJ was the main software used for this process.

### Tensile test and in vivo degradation

Sprague-Dawley rats (5-weeks-old; male: DBL) were used to test the tensile properties and to measure NGC degradation for sNGC, mNGC, and mspNGC (*n* = 3). SD rats were anesthetized with 2–3% isoflurane and the samples were implanted into the subcutaneous tissue. 4-, 8-, and 12 weeks post-surgery animals were euthanized via CO_2_ inhalation, the samples were explanted, and the surrounding tissues of NGCs were removed. The tensile test of samples was analyzed using the 5966 universal testing machine (5966 UTM). The tensile speed was 10 mm/min. NGC degradation was assessed based on the molecular weight of the samples. The explanted sample was dissolved in chloroform to separate the polymer from the surrounding tissue, and then precipitated with methanol. After dissolving the precipitated polymer in THF, residues were removed using a 0.2 μm filter, and the molecular weight was measured by GPC.

### Statistical analysis

All experimental results were statistically represented as the mean ± standard deviation except for tensile test. Experimental results of tensile test were represented as the mean ± standard error of the mean. Differences among samples were statistically assessed via two-way ANOVA, with Turkey’s analysis of variance using GraphPad Prism 8. Statistical significance was set at *p < 0.05 and **p < 0.01.

### Reporting summary

Further information on research design is available in the Nature Research Reporting Summary linked to this article.

## Supplementary information

Micropattern-based nerve guidance conduit with hundreds of microchannels and stem cell recruitment for nerve regeneration

REPORTING SUMMARY

## Data Availability

All data needed to evaluate are present in the paper and/or the Supplementary information. Additional data related to this paper may be requested from the authors.
