# Supplementary material for: Micropattern-based nerve guidance conduit with hundreds of microchannels and stem cell recruitment for nerve regeneration
Source: NPJ Regen Med. 2022 Oct 20;7:62. doi: 10.1038/s41536-022-00257-0 (PMC9582221; doi:10.1038/s41536-022-00257-0)
Supplement: Supplementary file 1 — Micropattern-based nerve guidance conduit with hundreds of microchannels and stem cell recruitment for nerve regeneration [file 41536_2022_257_MOESM1_ESM.pdf]

Supplementary Information

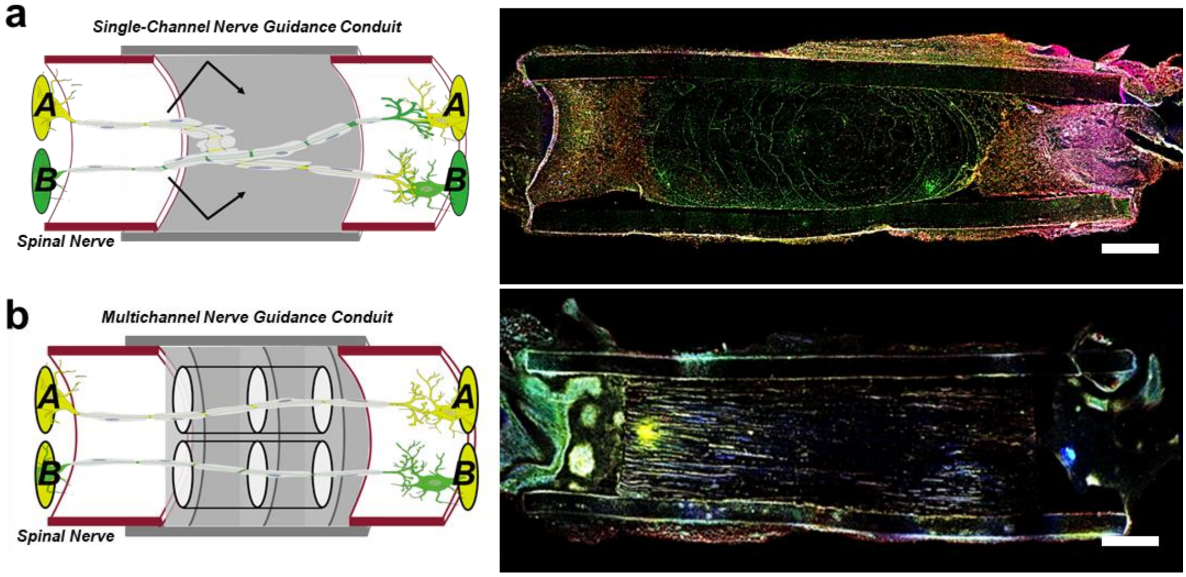

Supplementary Figure 1 Need for NGCs with multiple microchannels: (a) Possibility of incorrect reinnervation due to the absence of spatial guidance. (b) Role of the physical guidance for the alignment or correct reinnervation. Scale bar indicates 1 mm.

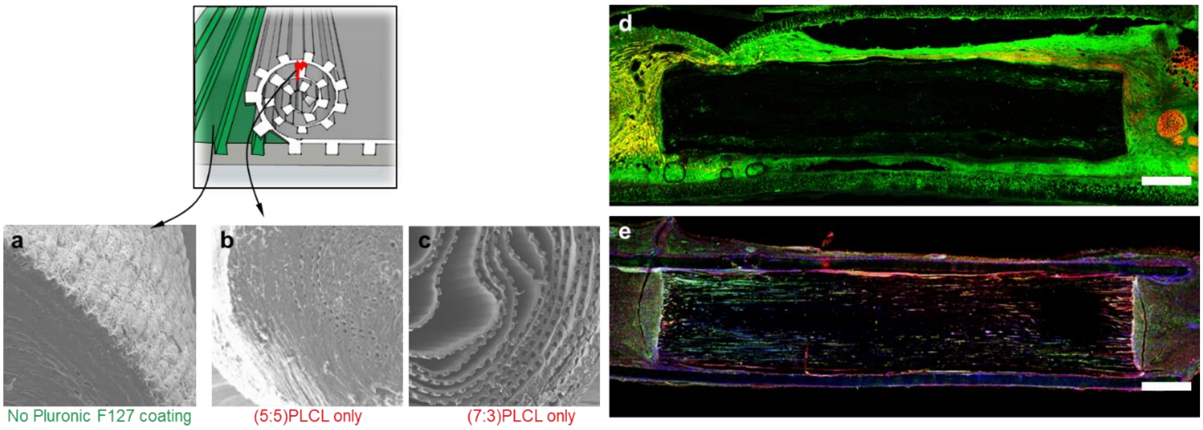

Supplementary Figure 2 Fabrication optimization for complete microchannel formation in mNGC: (a) Pre-coating with PF127 stops microbubble formation when the PLCL solution is spread on the patterned PDMS mold. (b) Collapse of the microchannels when using (5:5) PLCL only. (c) Unrolling disrupts the formation of microchannels when using (7:3) PLCL only. (d-e) Fluorescence micrographs of neuronal ingrowth into the mNGC microchannels with dimensions of (d) 10 μm × 10 μm × 20 μm and (e) 50 μm × 30 μm × 30 μm. Scale bar indicates (d-e) 1 mm.

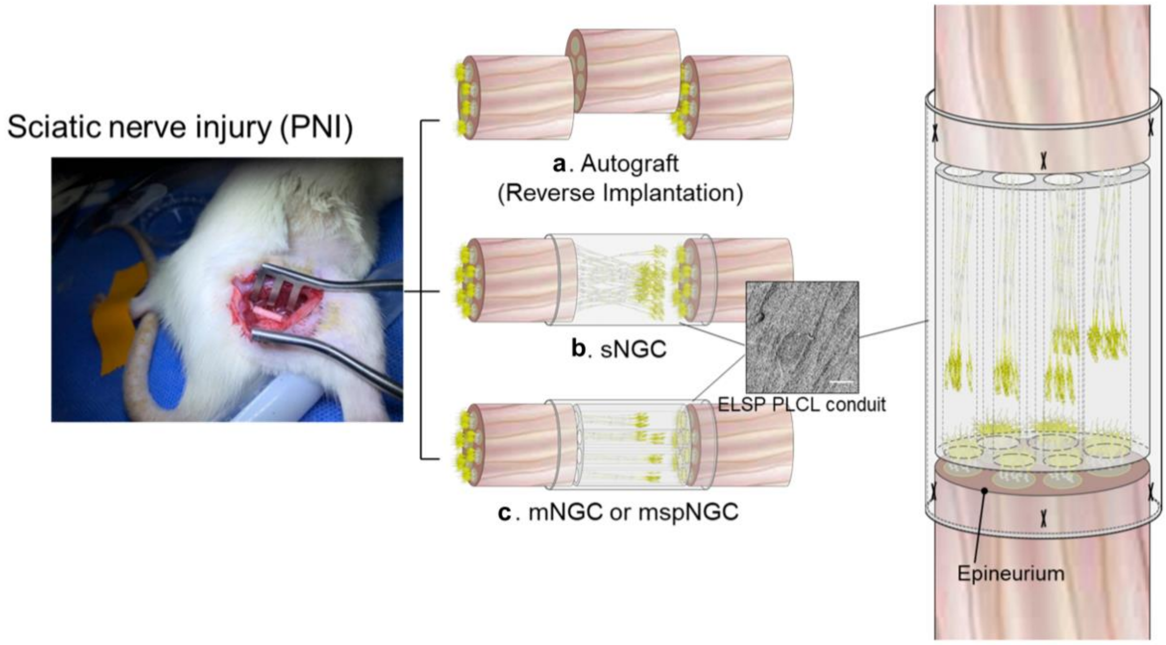

Supplementary Figure 3 Implants group in PNI model: (a) Autograft implant was the reverse of nerve tissue implant. (b) sNGC implant was the electrospun (ELSP) PLCL conduit. (c) mNGC or mspNGC affixed within the ELSP PLCL conduit were implanted into the injury. Scale bar indicates 100 µm in the subset image denoted 'ELSP PLCL conduit'.

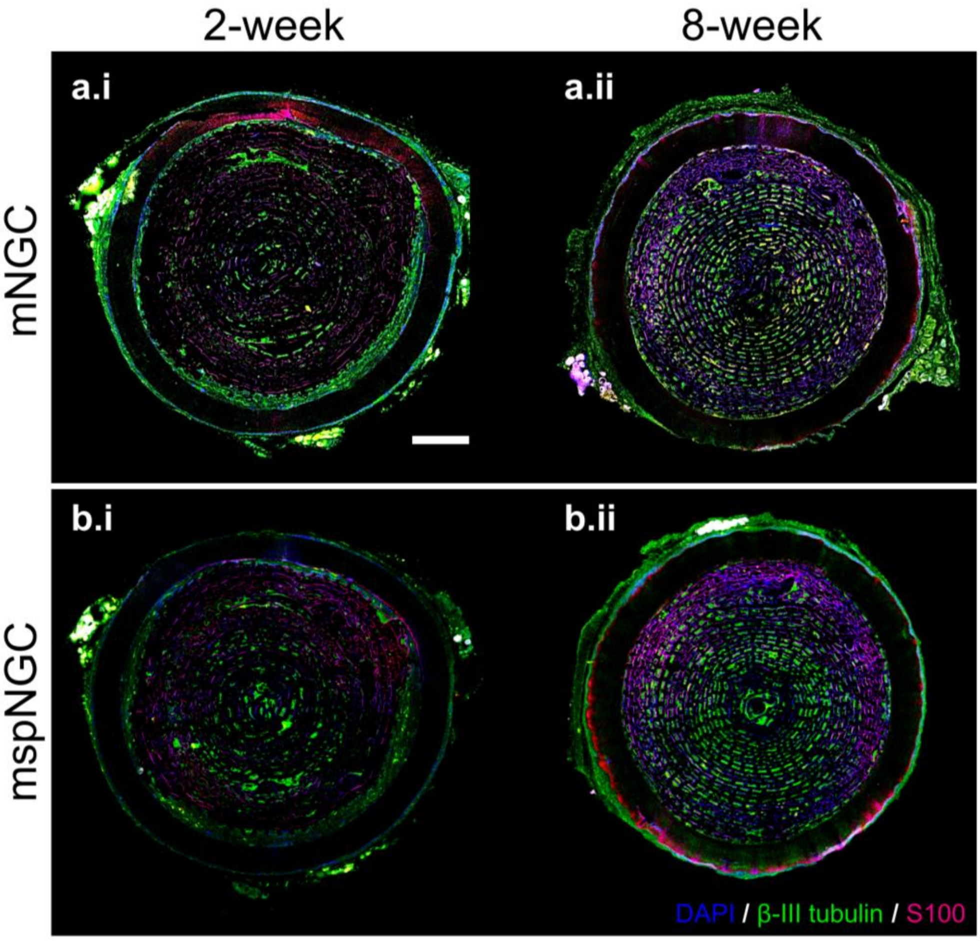

Supplementary Figure 4 Nerve regeneration mediated by the produced NGC: Fluorescence images of cross-sections at the middle of the (a) mNGC and (b) mspNGC. Green, red, and blue represent expressions of β-III tubulin, S100 and DAPI, respectively. Scale bar indicates 500 μm.

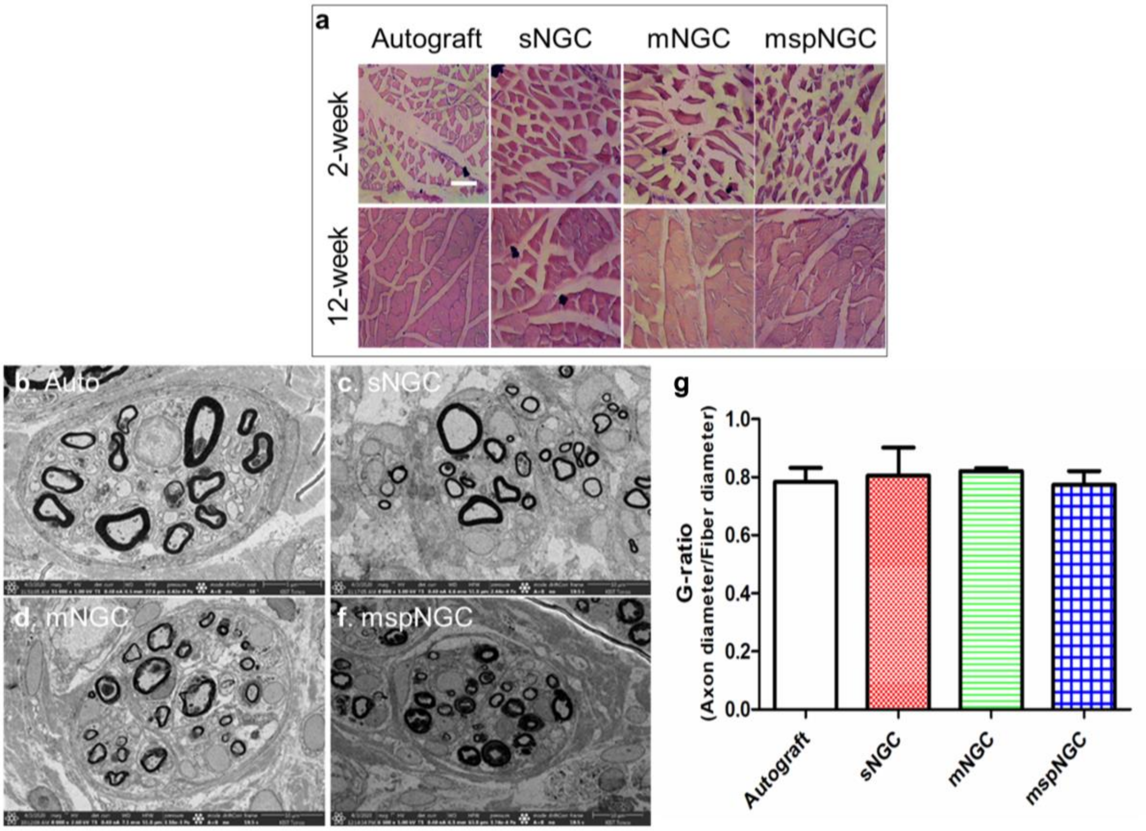

Supplementary Figure 5 Maturation of regenerated nerve fibers. (a) Optical images of the H&E stained gastrocnemius muscle tissue of each group at time points. SEM images of the planar cross-sections of (b) autograft, (c) sNGC, (d) mNGC, and (e) mspNGC implants, 8 weeks post-operatively. (f) G-ratios of the nerve fibers were analyzed to show the degree of maturation (n = 6 samples). Scale bar indicates (a) 100 μm. Values are presented as mean ± SD.

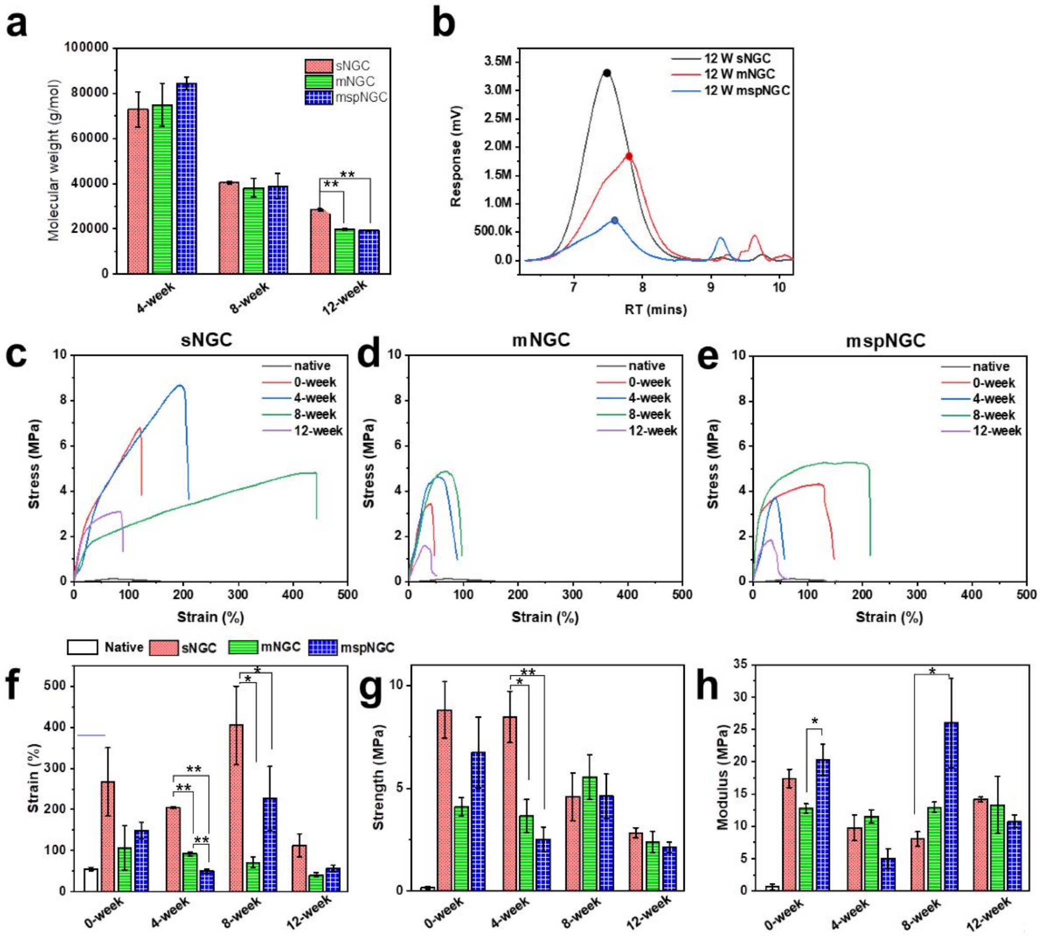

Supplementary Figure 6 In vivo degradation and mechanical properties of NGCs after implantation (n = 3) (a) Molecular weight of NGCs (***p* <0.01). (b) Molecular weight distribution poly of NGCs 12 weeks post-surgery. Tensile test of NGCs. Stress-strain curves of (c) sNGC, (d) mNGC, and (e) mspNGC. (f) Strain (**p* < 0.05; ***p* < 0.01), (g) strength (**p* < 0.05; ***p* < 0.01), and (h) modulus of NGC (**p* < 0.05). Values are presented as mean ± SEM.

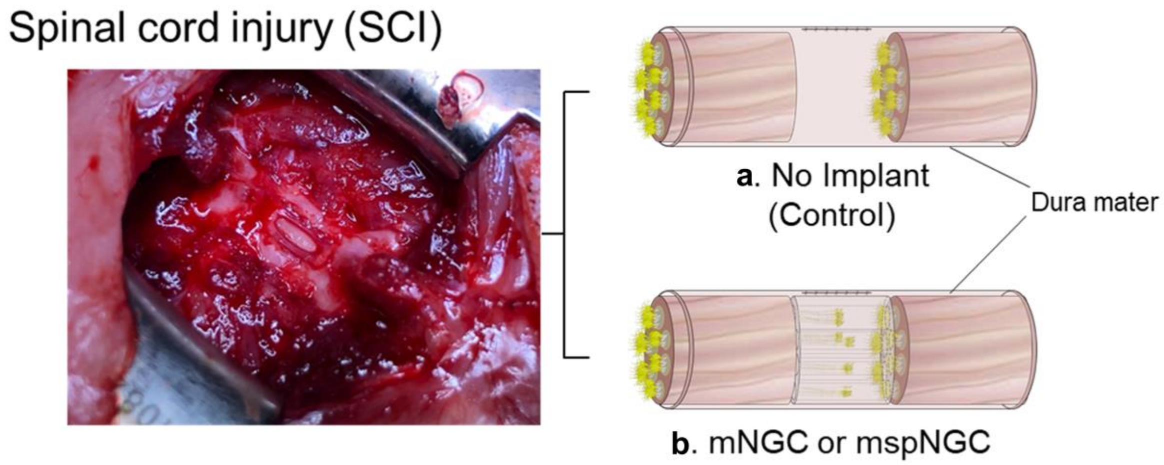

Supplementary Figure 7 Implant groups in SCI model: (a) Control group with no implants. (b) mNGC or mspNGC were implanted into the injury and were affixed within the dura mater.

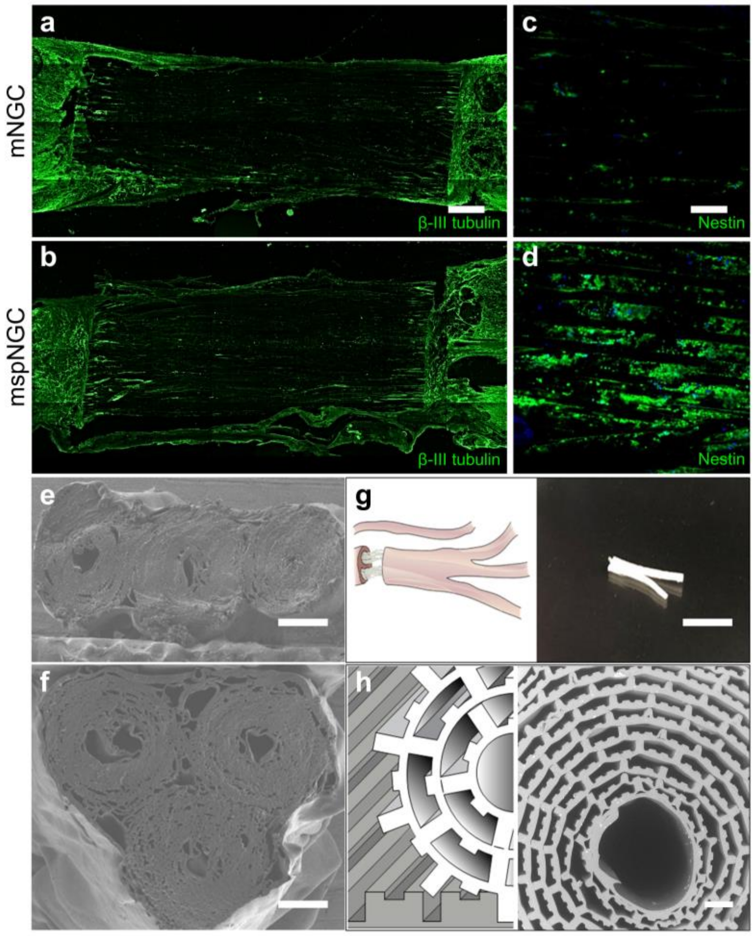

Supplementary Figure 8 Applicability in the SCI model and structural modification of the produced NGC. (a-d) Fluorescence micrographs of the longitudinal sections of the implant groups, showing the roles of (a, c) mNGC and (b, d) mspNGC in (a-b) guiding neuronal SCI regrowth (β-III tubulin) and (c-d) stem cell recruitment (Nestin). Possible modifications to the overall morphology of the NGC with three mNGCs (e) row or (f) pyramid stack, indicating its possible use for (g) a bifurcated tissue implant. (h) A schematic (left) and a micrograph (right) of a modified mNGC with augmented topological cues on the microchannel surface for enhanced cellular alignment. Scale bars indicate, (a, b) 50 μm, (c, d) 100 μm, (e, f) 200 μm, (g) 5 mm, and (h) 100 μm.

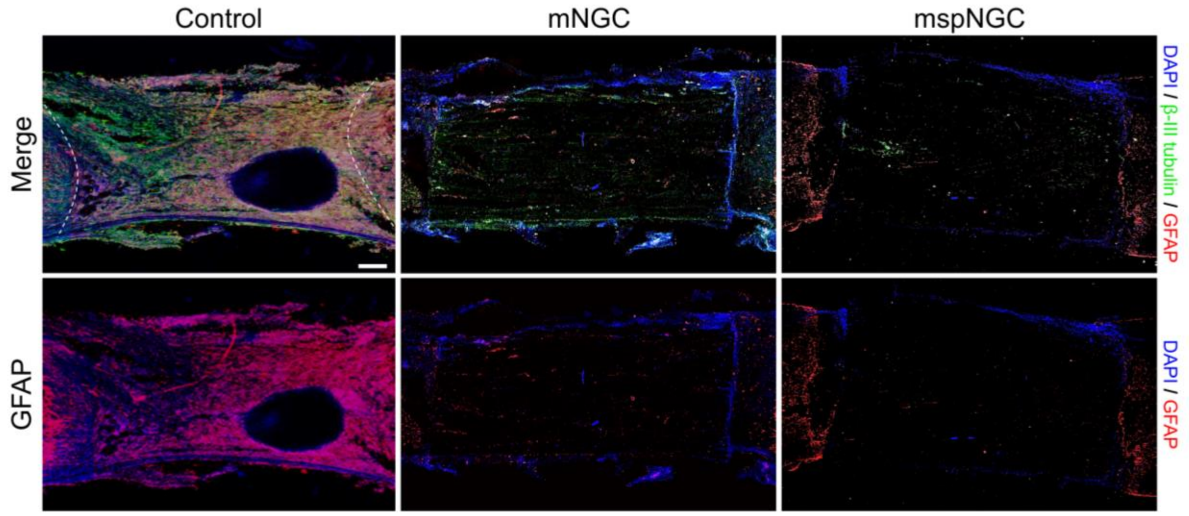

Supplementary Figure 9 Expression of neuronal cells and activated astrocytes in central nerve injury (CNI): Fluorescence micrographs of longitudinal sections of control, mNGC, and mspNGC groups at 2 weeks, showing neuronal regrowth and reactive astrocyte infiltration along the channels. Green, read, and blue represent the expressions of β-III tubulin, GFAP, and DAPI, respectively. Scale bar indicates 1 mm.
